# Movement patterns of a keystone waterbird species are highly predictable from landscape configuration

**DOI:** 10.1186/s40462-016-0092-7

**Published:** 2017-02-01

**Authors:** Erik Kleyheeg, Jacintha G. B. van Dijk, Despina Tsopoglou-Gkina, Tara Y. Woud, Dieuwertje K. Boonstra, Bart A. Nolet, Merel B. Soons

**Affiliations:** 1 Ecology & Biodiversity Group, Department of Biology, Utrecht University, Padualaan 8, 3584 CH Utrecht, The Netherlands; 2 Dutch Centre for Avian Migration and Demography, Netherlands Institute of Ecology (NIOO-KNAW), Droevendaalsesteeg 10, 6708 PB Wageningen, The Netherlands; 3 Department of Animal Ecology, Netherlands Institute of Ecology (NIOO-KNAW), Droevendaalsesteeg 10, 6708 PB Wageningen, The Netherlands; 4 Department of Zoology, University of Cambridge, Downing street, CB2 3EJ Cambridge, UK; 5 Theoretical and Computational Ecology, Institute for Biodiversity and Ecosystem Dynamics, University of Amsterdam, Amsterdam, The Netherlands

**Keywords:** *Anas platyrhynchos*, Connectivity, Dabbling ducks, Dispersal, Habitat fragmentation, Home range, Land use change, Mallard, Movement ecology, Telemetry

## Abstract

**Background:**

Movement behaviour is fundamental to the ecology of animals and their interactions with other organisms, and as such contributes to ecosystem dynamics. Waterfowl are key players in ecological processes in wetlands and surrounding habitats through predator-prey interactions and their transportation of nutrients and other organisms. Understanding the drivers of their movement behaviour is crucial to predict how environmental changes affect their role in ecosystem functioning. Mallards (*Anas platyrhynchos*) are the most abundant duck species worldwide and important dispersers of aquatic invertebrates, plants and pathogens like avian influenza viruses. By GPS tracking of 97 mallards in four landscape types along a gradient of wetland availability, we identified patterns in their daily movement behaviour and quantified potential effects of weather conditions and water availability on the spatial scale of their movements.

**Results:**

We demonstrate that mallard movement patterns were highly predictable, with regular commuting flights at dusk and dawn between a fixed day roost and one or several fixed nocturnal foraging sites, linked strongly to surface water. Wind and precipitation hardly affected movement, but flight distances and home range sizes increased when temperatures dropped towards zero. Flight distances and home range sizes increased exponentially with decreasing availability of freshwater habitat. Total shoreline length and the number of water bodies in the landscape surrounding the roost were the best predictors of the spatial scale of daily mallard movements.

**Conclusions:**

Our results show how mallards may flexibly adjust the spatial scale of their movements to wetland availability in the landscape. This implies that mallards moving between discrete habitat patches continue to preserve biotic connectivity in increasingly fragmented landscapes. The high predictability of mallard movement behaviour in relation to landscape features makes them reliable dispersal vectors for organisms to adapt to, and allows prediction of their ecological role in other landscapes.

**Electronic supplementary material:**

The online version of this article (doi:10.1186/s40462-016-0092-7) contains supplementary material, which is available to authorized users.

## Background

The spatiotemporal patterns of animal movement form an essential component of each species’ autecology, but are also fundamental elements of the ecology of the many species they interact with [1]. Individual movement trajectories, which collectively define an animal’s movement pattern, result from a combination of internal and external factors, including the animal’s motion capacity and the configuration of the landscape [2]. Studying the impact of these factors on animal movement is fundamental to understanding their role in the dynamics and functioning of natural ecosystems.

Highly detailed tracking data of birds are becoming increasingly available and have already provided a wealth of information supporting the development of species-specific population management schemes and protection plans (e.g. [3]). These data are also of critical importance to evaluate their interactions with other organisms, most importantly predator-prey interactions and transportation of nutrients and propagules. In a recent overview of ecosystem services provided by waterbirds, their role as highly mobile dispersers of aquatic invertebrates and plant seeds was identified as crucial for maintaining wetland biodiversity [4]. Equally relevant is the role of waterbirds in dispersing invasive species [5, 6] and pathogens, most notably avian influenza viruses (AIV [7, 8]). This transportation function is particularly relevant for dispersal of small flightless organisms between discrete and isolated habitat types, such as many wetlands [9, 10]. In order to assess the resilience of such landscape-scale ecological processes in the face of ongoing land conversion and habitat loss, it is crucial to understand the mechanisms underlying the movement patterns of keystone species and their response to environmental change.

Mallards (*Anas platyrhynchos*) are the most abundant and widespread duck species worldwide, with an estimated 19 million individuals across four continents [11, 12]. They are opportunistic habitat generalists, frequenting all wetland types, and often living close to humans in agricultural and urban areas [11, 13]. As such, they play a major role in wetland and terrestrial ecology, particularly as dispersal vectors of a wide variety of organisms including invertebrates [14, 15], plants [10, 16–19], and viruses, such as AIV [8, 20]. However, detailed studies of mallard movement patterns and the underlying mechanisms are scarce, hampering the assessment of the spatial scale of their contribution to ecological processes. While migration routes of mallards have recently been explored to evaluate their role in the continental spread of AIV [21, 22] and aquatic organisms [23, 24], only few studies have focused on day-to-day landscape-scale movement behaviour, with a strong bias towards North America (e.g. [25–28]). These studies, using radio telemetry, have shed light on various aspects of mallard movement ecology, revealing daily commuting behaviour and relatively small home range sizes, yet measured on a relatively coarse spatial and temporal resolution. Current high-resolution tracking techniques can provide much higher spatiotemporal detail of daily mallard movement patterns that allows analysis of time-activity budgets and behavioural strategies at the individual level across study sites [29–31].

Recent studies have shown that mallards may adjust their movement behaviour according to day length [29, 30], AIV infection status [20] and weather conditions [26, 29, 32], but variation among individuals is large. There is growing evidence that waterfowl densities and distributions during the non-breeding season are affected by landscape composition [31, 33, 34]. Although mallards are habitat generalists, they seem to depend heavily on surface water [27], so the availability of surface water in the landscape is likely a key factor driving their movements. We hypothesized that the affinity with water and persistent commuting behaviour of mallards across landscapes result in a negative relation between freshwater habitat availability and movement parameters (i.e. flight distance and home range size). Further, we hypothesized that weather conditions play an additive role in mallard movements, with reduced frequencies and distances under rainy, windy and cold conditions. To test these hypotheses, we performed a GPS telemetry study in four landscape types in the Netherlands, differing in the availability of surface water. First, we explored the daily movement patterns of mallards and verified their dependence on water. Then, we analysed the effects of weather conditions and landscape configuration on their spatiotemporal movement behaviour. Finally, we identified the specific relations between daily mallard movements and the availability of freshwater habitat from a compilation of mallard tracking studies from Western Europe to provide parameters to estimate the spatial scale of mallard movements based on simple landscape metrics.

## Methods

### Study sites

**Fig. 1:**
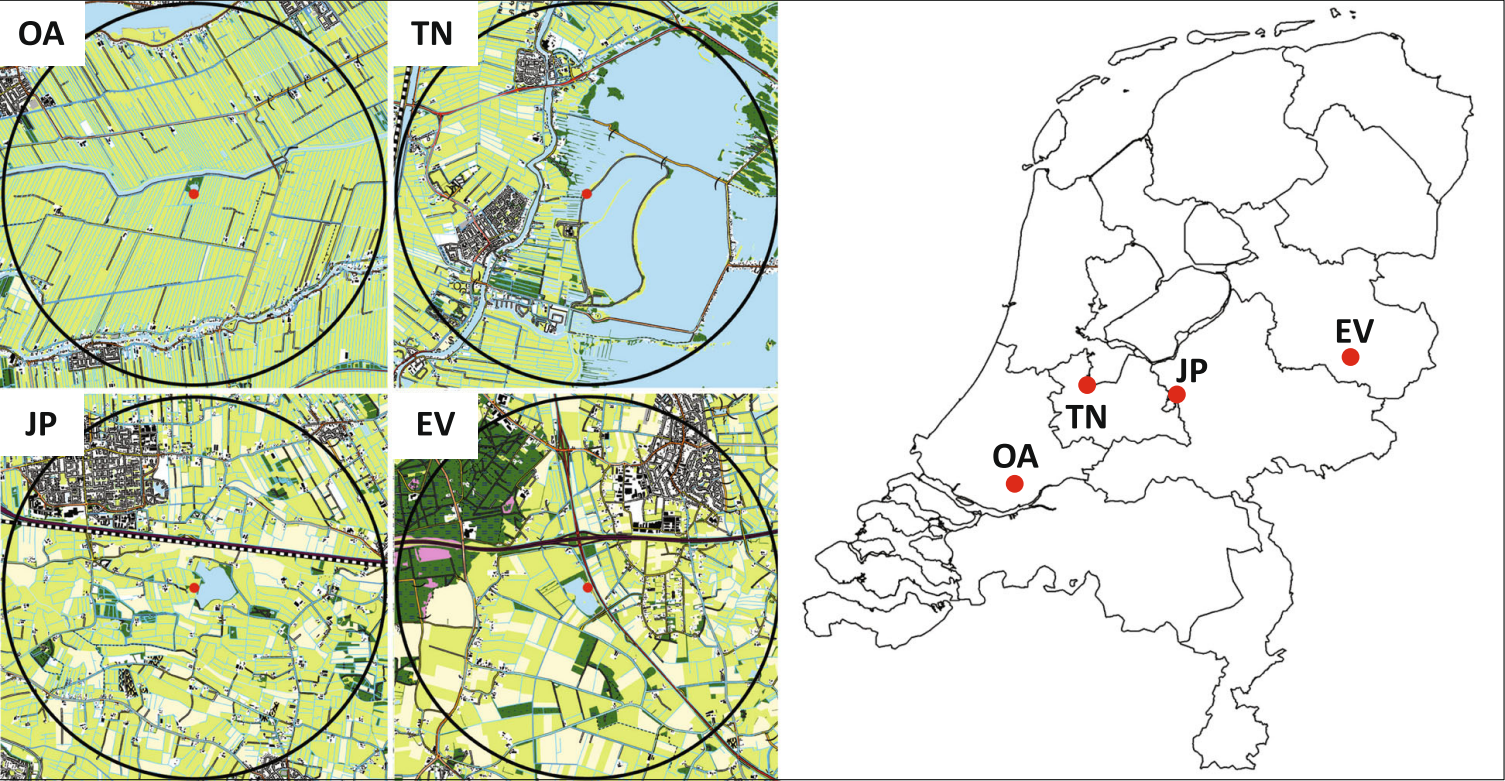
Topography and location of the four study sites in the Netherlands (OA = Oud Alblas, TN = Terra Nova, JP = Juliusput, EV = Enterveen). The vast majority (>96%) of observed mallard movements occurred within the *black circles* depicting the 2.5 km radius circle around the primary roost within which the landscape metrics have been calculated. The topographic maps show water (*light blue*), arable land (*yellow*), pastures (*light green*), forest (*dark green*), roads (*dark lines*) and buildings (*black dots*)

We selected four landscapes in the Netherlands varying in water availability along a west-east gradient from wet (peatland) to relatively dry (Pleistocene sand deposit) areas: Oud Alblas (51°52′35″N, 4°43′26″E), Terra Nova (52°12′55″N, 5°2′27″E), Juliusput (52°9′35″N, 5°28′44″E) and Enterveen (52°16′42″N, 6°33′32″E) (Fig. 1). Water bodies are plentiful in the Netherlands as it constitutes the delta of the rivers Rhine and Meuse and receives high annual precipitation (annual mean of 840 mm; Royal Dutch Meteorological Institute, www.knmi.nl), but from coastal to more inland areas there is a strong gradient in presence of surface water. The westernmost study site (Oud Alblas) lies 2 m below sea level, in a peat area with a dense network of narrow ditches (3–5 m wide) draining agricultural meadows, while the easternmost study site (Enterveen) lies 10 m above sea level, in a naturally-drained sandy soil region with scattered ponds and few canals (Fig. 1). Terra Nova lies around 0.5 m below sea level at the western end of a lake complex, close to a peat area similar to Oud Alblas. The Juliusput area lies at 4 m above sea level and has considerably less surface water than Terra Nova, but more small water bodies (Table 1). The four landscapes were selected to be similar in land use (mainly agricultural pastureland) and away from forest and built-up areas.

**Table 1:** Location, landscape characteristics and number of data points of the four main study sites in the Netherlands and the additional sites in the Netherlands, Switzerland (CH) and France (FR), used for the landscape metrics analysis across Western Europe

Location	Country	Latitude	Longitude	Water surface area (ha)	Number of water bodies	Total shore length (km)	Number of individual mallards	Number of duck days
Oud Alblas (OA)	NL	51.876505	4.723755	74.3	1335	362.5	73	1240
Terra Nova (TN)	NL	52.215404	5.040708	410.9	714	158.5	9	155
Juliusput (JP)	NL	52.159681	5.478855	56.2	1306	212.3	11	144
Enterveen (EV)	NL	52.278374	6.558803	29.4	596	115.7	4	85
Hendrik-Ido-Ambacht	NL	51.825879	4.647395	100.8	580	102.6	1	26
Almelo	NL	52.381949	6.625596	45.9	681	137.7	1	7
Ron	CH	47.172433	8.016636	18.6	25	40.1	1	-
Oberkirch	CH	47.162236	8.122171	768.4	79	66.2	8	-
Seine	FR	49.445392	0.372438	650.0	160	137.4	33	-
Brenne	FR	46.749784	1.233226	632.7	61	87.8	40	-

Swiss data are from [29] and French data from [26]. For the Swiss and French sites duck days were not available

### Catching and telemetry

At Oud Alblas, mallards were captured in a traditional duck decoy, a catching facility originally designed for commercial duck harvesting, but now often used for research [35]. At the three other sites, we used a mesh wire swim-in trap (LWH: 3.0 × 2.4 × 1.2 m) with funnel entrances on three sides, placed along the shoreline in *c*. 0.5 m deep water. All traps were placed in or near the main roost site in the area, based on the presence of mallards during the day. We provided bait (mixed grains) on small rafts inside the traps. We visited the traps every morning to retrieve mallards (and release occasional by-catches). When the traps were not used, feeding was continued but the funnels were removed to allow free passage of waterfowl.

Between 23 August 2012 and 21 March 2013, we captured 335 mallards. All were ringed, weighed, sexed and aged based on plumage characteristics [36] and biometrics were taken (maximum stretched wing, head + bill and tarsus length). Adult males (*N* = 164) were equipped with a CatTrack GPS logger (Catnip Technologies Ltd., Hong Kong) as a backpack using a Teflon harness (following [37]). Only adult males were used for GPS tracking to (i) avoid age-related variation, (ii) avoid the tracking of paired individuals with similar movement patterns [24], and (iii) minimize the relative weight of the loggers as males are heavier than females. The total weight of the package was 28 g, less than 3% of the male mallard body mass. The loggers were set to record a GPS position every 15 min until the battery ran out (estimated 14 days).

Since the GPS loggers did not provide remote data transfer, mallards were recaptured after several weeks using the same traps (63% success rate) to retrieve the logger and download the data. A breaking point in the Teflon harness ensured automatic logger detachment after several months in case recapture failed. We retrieved tracking data of 103 mallards, but battery life and time interval accuracy varied between loggers. To deal with different tracking durations between individuals, we broke down the data into ‘duck days’: 24 h periods from noon to noon, including the entire nocturnal active period. The number of sampled duck days per individual varied from 2 to 31 (mean 18 days). We removed all duck days with less than 70 fixes (mean number of fixes minus one standard deviation) per day, keeping only high quality trajectories with regular GPS intervals of 15–20 min. After this selection, the tracks of 97 individual mallards remained, covering 1624 duck days (1240, 155, 144 and 85 duck days over 73, 9, 11 and 4 individuals at Oud Alblas, Terra Nova, Juliusput and Enterveen, respectively).

### Movement parameters

From the GPS positions we calculated the following movement parameters: flight frequency, flight distances (of individual flights, mean per day and maximum per day), home range size, core area size and the number of core areas.

First, we detected and deleted erroneous outlying GPS fixes. Mean GPS error was 6.4 m (±8.4 SD) based on 9451 GPS positions of three loggers fixed to a pole. Obvious large errors (‘spikes’) were defined as apparent displacements of >100 m with a direct return at least halfway back to the original position (1.4% of the positions) and were deleted before further analysis. After this purge, we identified individual flights. Movements within foraging or roosting sites rarely exceeded 100 m in 15 min, although mallards could easily cover this distance when walking or swimming in a straight line [38]. Especially at Oud Alblas, most flights (switches between core areas) were too short to use the threshold distance of 250 m between GPS fixes as suggested in an earlier study in North America [29]. Therefore, we defined displacements of >100 m between two GPS positions as flights. That way, some long swimming or walking bouts may have been mislabelled as flights, but patterns of flight frequency and flight distance were similar when using alternative thresholds of 250 m and 450 m (Additional file 1: Figure S1). Other movement metrics were independent of the definition of flights. Consecutive displacements of >100 m (i.e. when a GPS position was recorded during flight) were merged, with recalculation of the flight distance between the first and last position of the total displacement. Flight frequencies and flight distances (great circle distance) were calculated from individual flights. Circadian patterns in flight behaviour were analysed by separating the day in four periods: day (between sunset and sunrise), dusk (the 1.5 h after sunset), dawn (the 1.5 h before sunrise) and night (between dusk and dawn). This distinction of day periods was based on the distribution of flights over the day (Additional file 1: Figure S2).

To identify the dependence of mallards on water, we calculated the shortest distance to water from each recorded GPS position using high-resolution topographic maps of water bodies (BRT Top10NL 2013, Kadaster Nederland) in ArcGIS, and compared this against the distance to water from 1000 random points in the landscape within a 2.5 km radius circle around the roost (depicted in Fig. 1).

Finally, we calculated daily movement parameters representing the spatial scale of mallard landscape use. Home range size per duck day, i.e. the total area traversed by a mallard including flights, was calculated as the area of daily minimum convex polygons (MCP) including 100% of the recorded GPS positions. The size of the most intensively used areas in the landscape per duck day (core area size, excluding flights) was calculated as the area of the 50% kernel utilization density (KUD) (R-package adehabitatHR [39]). To determine the number of core areas visited per duck day, we determined core area locations by averaging the latitudes and longitudes of GPS positions between two flights. If the distance between these central points exceeded 450 m (the distance that mallards could swim within 15 min without substantial effort [38]), they were considered separate core areas.

### Weather and landscape parameters

Hourly weather data were obtained from the Royal Dutch Meteorology Institute (www.knmi.nl). For each study site, we used weather data from the nearest weather station: Cabauw for Oud Alblas, De Bilt for both Terra Nova and Juliusput, Heino for Enterveen, all within 30 km distance from the main roost. We calculated the mean temperature, mean wind speed and total amount of precipitation for each duck day.

We identified the availability of surface water in each study area using high-resolution topographic maps (BRT Top10NL 2013 Kadaster Nederland) of the different landscapes in the Netherlands. Within a 2.5 km radius circle around the trap locations (which fully included 96% of all flights and 94% of all GPS positions, depicted in Fig. 1) we calculated the following parameters of surface water availability: 1) water surface area, 2) the number of separate water bodies, and 3) total shore length. These landscape metrics are the simplest predictors of freshwater habitat availability in a landscape and can be calculated from any high-resolution topographic map, facilitating extrapolation of our results to other landscapes. Short excursions where mallards used a roost outside of the study landscape for one or two days occurred on four occasions (once at Terra Nova and Enterveen, twice at Juliusput) and were excluded from the landscape analysis since these movements were subject to different landscape configurations.

### Additional movement data

We complemented our dataset with additional mallard tracking data from landscapes with different wetland availabilities. Firstly, we included the data of two birds that had left the study landscapes for a longer period of time and moved to respectively Hendrik-Ido-Ambacht (51°49′33″N, 4°38′51″E, 26 days) and Almelo (52°22′55″N, 6°37′32″E, 7 days). Secondly, we included data from two study sites in France (Seine 49°26′43″N, 0°22′21″E and Brenne 46°44′59″N, 1°13′60″E; [24]) and Switzerland (Ron 47°9′44″N and Oberkirch 8°7′20″E and 47°10′21″N, 8°0′60″E; [27]). Due to limited data from these sites, we calculated movement metrics at the level of individual mallards rather than duck days, and recomputed the same metrics for the mallards of our tracking study in the Netherlands. For the French study, which provided only mean home range sizes and flight distances of multiple individuals, we used the mean, standard deviation and sample size to construct normal distributions from which we drew values per individual mallard for the analysis. Landscape metrics of the French and Swiss areas were calculated using OpenStreetMaps, supplemented with satellite images to match the resolution of the topographic maps of the Netherlands.

### Statistical analyses

After first exploring mallards’ general movement behaviour (Additional file 1: Figure S3), we analysed the effects of weather conditions and study site on their movement patterns across the four study landscapes in the Netherlands, for which most (detailed) data was available. Subsequently, we explicitly analysed the associations between mallard movements and landscape metrics at all study sites in the Netherlands, Switzerland and France.

To explore the general movement behaviour irrespective of landscape or study site, we evaluated circadian patterns in flight behaviour and habitat use. We used a linear mixed-effects model (LMM; model 1 in Table 2) to test how flight behaviour (quantified as distances covered by individual flights) was affected by the time of the day (TOD: day, dusk, night and dawn) and weather conditions (wind, precipitation and temperature) measured during the hour in which the flight occurred, using study site and individual mallard as random factors. Due to collinearity of weather parameters, we introduced the first two principal components of a PCA on wind, precipitation and temperature (together explaining 98.8% of variation) in the model instead of the original data. The first component (PC1) correlated strongly with temperature (r_s_ = −0.99, *p* < 0.001), and the second component (PC2) correlated with wind (r_s_ = −0.69, *p* < 0.001) and precipitation (r_s_ = −0.67, *p* < 0.001). We selected the best fitting models based on Akaike Information Criterion corrected for small sample size (AICc), with the best fitting model having the lowest AICc value, and candidate models with a difference in AICc (ΔAICc) of <2 considered equivalent [40]. We further explored the general movement behaviour by evaluating whether the distance to open water from the mallards’ GPS positions differed from random points in the landscape and whether this pattern differed between night and day using ANOVA with Tukey HSD post-hoc tests.

**Table 2:** Summary table showing AICc, ΔAICc and Akaike weight (ω_i_) values of linear mixed-effects models explaining variation in movement variables

Model	Movement parameters	Independent variables	AICc	ΔAICc	ω_i_
(1)	Flight distance	**~** TOD + PC1	16394	0.00	0.65
		**~ TOD**	**16396**	**1.49**	**0.31**
		~ TOD + PC1 + PC2	16401	6.77	0.02
		NULL	17705	1310.67	0.00
(2)	Number of flights	**~ study site + PC1**	**6790**	**0.00**	**0.73**
		~ study site + PC1 + PC2	6792	1.99	0.27
		~ study site	6804	13.90	0.00
		NULL	6875	85.44	0.00
	Mean flight distance	**~ study site + PC1**	**4520**	**0.00**	**1.00**
		~ study site + PC1 + PC2	4531	11.32	0.00
		~ PC1	4542	22.55	0.00
		NULL	4552	32.43	0.00
	Maximum flight distance	**~ study site + PC1**	**4845**	**0.00**	**1.00**
		~ study site + PC1 + PC2	4856	10.94	0.00
		~ study site	4873	27.87	0.00
		NULL	4890	44.95	0.00
	Home range size	**~ study site + PC1**	**4009**	**0.00**	**1.00**
		~ study site + PC1 + PC2	4023	13.53	0.00
		~ study site	4033	23.57	0.00
		NULL	4060	50.47	0.00
	Core area size	**~ study site + PC1**	**1032**	**0.00**	**0.99**
		~ study site	1041	8.71	0.01
		~ study site + PC1 + PC2	1047	14.83	0.00
		NULL	1068	35.79	0.00
	Number of core areas	~ study site + PC1	4252	0.00	0.20
		~ PC2	4252	0.02	0.20
		NULL	4253	0.43	0.16
		~ PC1	4253	0.85	0.13
(3)	Maximum flight distance	**~ surface + shore length**	**299**	**0.00**	**0.65**
		~ surf. + water bodies + shore l.	301	1.83	0.26
		~ shore length	304	5.52	0.04
		NULL	314	15.50	0.00
	Home range size	**~ shore length**	**480**	**0.00**	**0.47**
		~ surface + shore length	481	1.48	0.23
		~ water bodies + shore length	481	1.51	0.22
		NULL	499	19.37	0.00

Model set 1 concerns the analysis of individual flights, model set 2 concerns the analysis of movement metrics per duck day in the Netherlands, and model set 3 concerns the analysis across Western Europe. Only the top three and null models are shown (full details in Additional file 1: Table S2). Best fitting models without uninformative parameters [49] are in bold. TOD is time of day (sunrise, day, sunset or night), and PC1 and PC2 are principal components of weather parameters, mainly corresponding to temperature and wind/precipitation, respectively. Flight distance and home range parameters were log-transformed

Then, we tested how movement parameters per duck day were affected by weather conditions and study site using LMMs, using mallard ID and date as random factors (model 2 in Table 2). For the number of flights and number of core areas (i.e. count data), we used models assuming a Poisson error distribution with a log-link function. We used the same procedure for model selection as described above. Here, we again analysed effects of weather by introducing the two principal components (together explaining 94.6% of variation) of a PCA on the weather parameters in the model. Again, PC1 correlated most strongly with temperature (r_s_ = −0.93, *p* < 0.001) and PC2 correlated with wind (r_s_ = −0.28, *p* < 0.001) and precipitation (r_s_ = −0.67, *p* < 0.001).

Finally, we tested the associations between mallard movements and landscape configuration for all study sites in the Netherlands, Switzerland and France. We used LMMs to test the relations of mean daily maximum flight distance (a proxy for the distance between roost and foraging sites) and home range size per individual mallard with the landscape metrics (water surface area, the number of separate water bodies, and the total shore length). Landscape metrics were introduced in the models as fixed effects, using study site and country (i.e. original tracking study) as random factors (model 3 in Table 2). Due to high correlation between the number of water bodies and total shore length (r_s_ = 0.95, *p* < 0.001; Additional file 1: Figure S4), we focused this analysis on univariate models including each landscape parameter separately (Table 3).

**Table 3:** Statistics of linear mixed-effects models to test the effects of landscape metrics on the maximum flight distances between roost and foraging sites and home range sizes of mallards in Western Europe

	Dependent variable (log-transformed)	Fixed effect	Intercept (±SE)	Estimate	SE	*p*-value	Marg. R^2^	Cond. R^2^
A	Maximum flight distance	Water surface area	8.41 (0.33)	−0.00118	0.00043	<0.001	0.29	0.47
		Total shore length		−0.00521	0.00077			
B	Maximum flight distance	Water surface area	7.77 (0.52)	−0.00113	0.00085	0.344	0. 11	0.71
		Number of water bodies	7.69 (0.43)	−0.00104	0.00046	0.074	0.33	0.76
		Total shore length	7.73 (0.23)	−0.00436	0.00069	0.001	0.41	0.58
	Home range size	Water surface area	5.73 (0.51)	0.00097	0.00116	0.329	0.03	0.41
		Number of water bodies	6.82 (0.39)	−0.00145	0.00049	0.009	0.29	0.45
		Total shore length	7.55 (0.20)	−0.00989	0.00074	<0.001	0.54	0.54

Test results are shown for the top model for maximum flight distances, containing both water surface area and total shore length (A), and for univariate models for maximum flight distance and home range size (B). *P*-values were computed using likelihood ratio tests between the null models and the models containing the metric of interest as only independent variable. Marginal and conditional R^2^ values were calculated following [52]

For all statistical tests, flight distance, home range size and core area size values were log-transformed to approach normality of the residuals. We performed Tukey HSD post-hoc tests on the effects of TOD and study sites. Statistical tests were performed using the packages lme4 [41] and multcomp [42] in R [43].

## Results

### General movement patterns

**Fig. 2:**
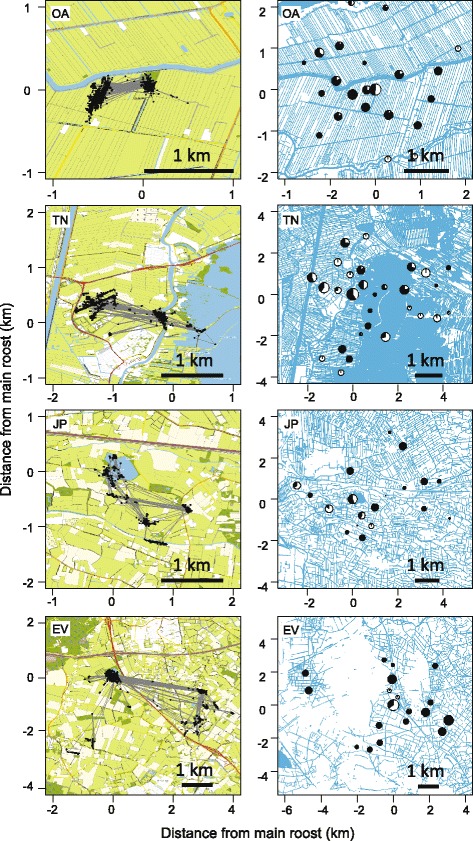
Example of representative tracks of a single mallard per study landscape (*left*; ca. 15 days per track) and the use of core areas in the landscapes of all tracked individuals separated between day (*light parts in pie charts*) and night (*dark parts*; *right panels*). The size of the pie charts is scaled to the relative number of GPS fixes recorded in the core areas. OA = Oud Alblas, TN = Terra Nova, JP = Juliusput, EV = Enterveen. Note the differences in scale: mallards at Oud Alblas use a much smaller part of the landscape around the main roost than do mallards at Enterveen. Left panels are zoomed in on the individual tracks. The maps show water (*light blue*), arable land (*yellow*), pastures (*light green*), forest (*dark green*), roads (*dark lines*) and buildings (*black dots*)

Throughout the study period and across all study sites, the spatiotemporal patterns of daily mallard movements showed high similarity and predictability. Most individuals showed high site fidelity, repeatedly visiting the same diurnal roost and nocturnal foraging sites (Fig. 2). On 92.8% of days mallards used the same roost as the day before, and on another 4.7% of days, a roost was visited that had been used by the individual earlier within the tracking period. The probability of revisiting a familiar foraging site was 96.4% per night, while mallards explored new foraging sites (not visited before during the tracking period) on only 12.4% of nights. Fifty-six mallards (58%) used a single day roost during the entire tracking period, particularly at Oud Alblas. The day roost was usually one of the larger water bodies, where most mallards in the area gathered. However, at all study sites several individuals occasionally used alternative roosts (Fig. 2). At night, the mallards usually visited smaller water bodies such as narrow drainage canals (ditches) and streams for foraging.

Flights were mostly concentrated around sunset and sunrise, corresponding to the circadian pattern of core area use. We observed 37.8% of all flights during dusk and dawn (dusk: 18.6%, dawn: 19.2%), irrespective of seasonal changes in day length (Additional file 1: Figure S2). Flight distances did not differ between dusk and dawn (Tukey HSD post-hoc test; *z* = 1.0, *p* = 0.730), but were almost twice as long as during the day (*z* = 30.1, *p* < 0.001, and *z* = 31.0, *p* < 0.001 respectively) and approximately 60% longer than during the night (*z* = 22.2, *p* < 0.001, and *z* = 23.5, *p* < 0.001, respectively). Nocturnal flights were on average 26% longer than daytime flights (*z* = 11.0, *p* < 0.001; Additional file 1: Figure S2). The longest flight recorded during this study was 25.5 km of a bird leaving its home range, while the overall mean flight distance was only 0.4 km (Additional file 1: Table S1, Additional file 1: Figure S3a) and the average longest flight per day was 0.9 km (Additional file 1: Figure S3b). Besides movements during dusk and dawn, mallards switched core areas 2.6 times more often during the night than during the day. On 88.4% of the duck days, the mallards did not leave the roost during the day and in 67.0% of the nights mallards used only one foraging site. The maximum number of different core areas visited per night was 6 (at Juliusput).

Unlike behavioural patterns, home range sizes and displacement distances varied substantially between individuals (Additional file 1: Figure S3). The daily home range size within which all displacements occurred (100% MCP), ranged between 0.3 and 1416.9 ha, although the median of 9.7 ha per duck day indicates that most animals stayed in a very restricted area (Additional file 1: Table S1, Additional file 1: Figure S3c). The size of the area that was used most intensively by mallards (50% KUD), was on average 3.9% of the total home range and had a median of 0.3 ha per day (range 0.03–40.9 ha) (Additional file 1: Table S1, Additional file 1: Figure S3d). On average, mallards visited two core areas per day (Additional file 1: Figure S3e) and performed 4–7 flights per day (Additional file 1: Table S1, Additional file 1: Figure S3f). Daily 100% MCP home range size was obviously strongly correlated with daily mean and maximum flight distances (r_s_ = 0.74 and 0.88 respectively, both *p* < 0.001).

### Affinity for water

**Fig. 3:**
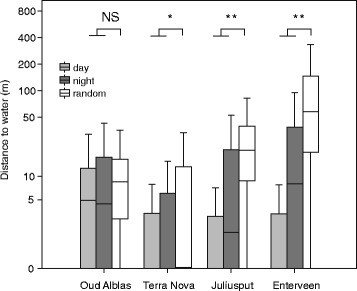
Distance to water in different landscapes during daytime and night time compared to 1000 random points in the same landscape. The boxplots show the median with 25th and 75th percentiles and error bars represent the range of 95% of all positions. Significance levels of differences between GPS positions and random points are depicted (* *p* < 0.05; ** *p* < 0.01). Note the log scale of the y-axis

The mallards showed strong affinity for water at all study sites, both during roosting and foraging (Fig. 3). Overall, 95% of all GPS positions per mallard were within 38 m from the nearest water body. Nocturnal positions in all landscapes were on average farther away from water (mean = 11.4 m, 95th percentile = 44.4 m) than diurnal positions (mean = 6.7 m, 95th percentile = 23.2 m). However, even during nocturnal foraging, the absolute distances to the nearest water body remained short and in all study sites except Oud Alblas the nocturnal positions were still significantly closer to water than random (Fig. 3). Occasional foraging on agricultural fields without water in the direct vicinity was only observed at the driest study site, Enterveen, suggesting lower dependence on water bodies when foraging in drier landscapes.

### Effects of weather and study site on movement patterns

Flights occurred under a wide range of weather conditions, with temperatures ranging between −12.5 and +26.7 °C, wind speeds up to 14 m/s and highest hourly precipitation of 8.7 mm. Wind speed and precipitation (PC2) did not contribute to the best model fit for any of the mallards’ movement parameters, except for a marginal additive contribution to the number of flights per day (ΔAICc = 1.99). In contrast, temperature (PC1) contributed to each of the top models and explained part of the variation for each movement parameter, except the number of core areas (Table 2, Additional file 1: Table S2). For the number of core areas, there was no model fit better than the null model.

**Fig. 4:**
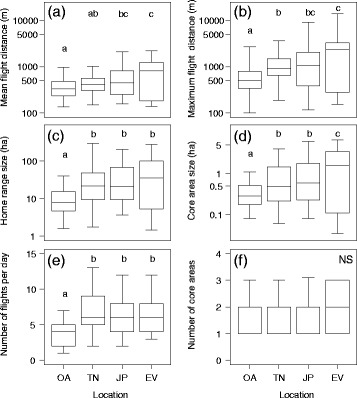
The spatial scale of mallard movements at the four study sites in the Netherlands (OA = Oud Alblas, TN = Terra Nova, JP = Juliusput, EV = Enterveen) in the order of high to low freshwater habitat availability. Graphs represent **a** the mean flight distance per day, **b** the maximum flight distance per day, **c** the total home range size (100% MCP), **d** the core area size (50% KUD), **e** the number of flights per day, and **f** the number of core areas visited per day. The boxes indicate the median between 25th and 75th percentiles with whiskers depicting the 5th and 95th percentiles. Letters denote statistical differences (*p* < 0.05) based on HSD Tukey post-hoc tests following linear mixed-effects models (Table 2)

Study site contributed to the best model fits for all movement parameters, again except the number of core areas. Movement parameter values at the site with the highest availability of shoreline habitat (Oud Alblas) were consistently lower than in all three other landscapes, with the single exception of mean flight distance at Terra Nova, the site with most surface water (Fig. 4). Mean daily maximum flight distances increased from 0.6 ± 0.5 km at Oud Alblas to 2.1 ± 2.0 km at Enterveen, the driest site, with intermediate values at Terra Nova and Juliusput. Similarly, home range size increased from 14.0 ± 28.7 ha at Oud Alblas to 91.3 ± 184.2 ha at Enterveen, and core area size from 0.4 ± 0.7 to 2.7 ± 5.0 ha (Fig. 4).

### Effects of landscape configuration on movement patterns

**Fig. 5:**
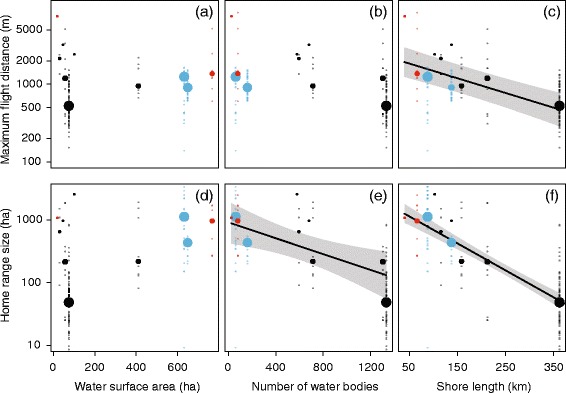
Relations between the movement parameters flight distance between roost and foraging site (**a**-**c**) and home range size (**d**-**f**) with the landscape metrics water surface area (**a**, **d**), total number of water bodies (**b**, **e**) and total shore length (**c**, **f**) within a 2.5 km radius around the primary roost. Data from study areas in the Netherlands (*black*), Switzerland (*red*), and France (*blue*) are shown. Solid points depict median values per landscape (with sizes proportional to the logarithm of corresponding sample size), transparent points represent original data, and *lines* and *shaded areas* depict the fitted line with 95% confidence intervals for the landscape metrics significantly contributing to log-transformed movement parameters (Table 3). Note the log-scale of the y-axis

Analysis of the extended Western European dataset showed that the top models explaining maximum flight distance and home range size included a combination of landscape metrics. The top-ranking parsimonious model explaining variation in home range size contained total shore length as the single best predictor. In contrast, maximum flight distances were best explained by both shoreline length and water surface area (Table 2). However, total shore length was the major explanatory variable here, as it was also strongly related to flights when tested separately, while water surface area was not (Table 3). This indicates that water surface area was only significant in explaining residual variance in the model containing total shore length, and only plays an additional explanatory role after the effects of total shore length are accounted for. When focusing on models containing a single landscape parameter as explanatory variable, we found that only total shore length explained variation in maximum flight distance significantly better than the null model (ΔAICc = 10.0, *p* = 0.001; Table 3, Fig. 5). As single predictors explaining variation in home range size, both total shore length (ΔAICc = 19.4, *p* < 0.001) and the number of water bodies (ΔAICc = 4.7, *p* = 0.009) performed better than the null model (Table 3, Fig. 5).

## Discussion

This study demonstrates that the spatial scale of mallard movements is variable between localities, and highly predictable from the availability of freshwater habitat in the landscape. Despite being known as a habitat generalist [11, 13], mallards appear to select their habitat strongly based on the presence of open water, both during day and night, suggesting that water availability in the landscape should affect their movements. While temperature consistently appeared as an additive effect explaining variation in movement behaviour at the four study sites in the Netherlands, study site had the strongest effect, with differences in water availability as most likely underlying mechanism. This hypothesis was further supported by the strong effects of total shore length and, to a lesser extent, the number of waterbodies, on the scale of mallard movements across Western Europe.

### General movement patterns

Commuting behaviour between a limited number of fixed diurnal roost and nocturnal foraging sites is a common phenomenon among dabbling ducks [26, 44–46]. This has been reported also for mallards, in Europe and North America [28, 30], although some suggest that mallards are more flexible due to their broad habitat requirement and therefore have a more variable landscape use [27]. We found that, despite their flexibility, mallards across a wide range of landscapes have very high site fidelity and strongly select for habitat close to open surface water. Within each studied landscape in the Netherlands, there was one main roost that seemed to attract most mallards in the area, typically a relatively large water body with vegetated shores providing cover. From there, the birds spread out over the surrounding landscape at dusk, towards smaller and more exposed water bodies. This suggests that these landscapes were used as if composed of “functional units”, a concept introduced by Tamisier [44], stating that foraging sites in an area are used by ducks from a single, central roost, and that a region contains multiple functional units without overlap. Support for this concept was found in several dabbling duck species in Europe, Africa and North America, but Tamisier recognized that the case of mallards could be more complex, due to their ecological plasticity [44]. Indeed, within our study landscapes multiple smaller roost sites were used regularly, and individuals from different roosts used overlapping foraging sites. Moreover, some areas were frequented during both day and night (Fig. 2). Almost half of the mallards used more than one roost site during the tracking period of 18 days on average. The use of alternative roost sites seemed to occur most frequently in the wettest landscapes (79% of all core areas were used also during daytime hours at Oud Alblas and 68% at Terra Nova, versus 42% at Juliusput and 36% at Enterveen, Fig. 2). This might suggest that congregation of ducks at a single roost in the landscape reflects limited availability of suitable or undisturbed habitat, rather than an obligatory behavioural trait.

Due to the necessity to recapture mallards for data retrieval, mallards with commuting behaviour might be overrepresented in our dataset, but birds that were shot or recaptured outside of the studied landscapes did not behave differently. Mallards that left the study area (and of which GPS data were retrieved; *N* = 5), directly resumed their commuting behaviour from a central roost upon arriving in a different landscape. Interestingly, recapture rates were lowest at Enterveen, the driest study site in the Netherlands, and ring recoveries of the GPS tracked mallards later revealed that the proportion of (long-distance) migrants was probably highest at this site (data not shown). This raises the question whether migratory mallards show less site fidelity during the non-breeding season than year-round residents, but our data does not allow for such a comparison.

From a connectivity perspective, the movement patterns we describe imply that mallards selectively use only parts of the landscape. It suggests that their capacity to disperse other organisms is highly predictable and may have resulted in the evolution of specific dispersal traits to promote dispersal by mallards, particularly in species with a similar affinity for water, as already hypothesized by Darwin [9]. Such traits have indeed been proposed for aquatic invertebrates such as aquatic snails [15], but have not been confirmed for plant seeds [47]. Considering dispersal of plants, a wide range of species associated with aquatic and terrestrial habitats are involved [17] and these are likely to be dispersed across a range of inundated to upland conditions found in the direct vicinity of water bodies. For the transmission and spread of infectious diseases such as AIV, the combination of daytime clustering and night-time scattering of mallard individuals seems highly effective and this may be one explanation why infection rates in waterbirds are generally high (e.g. [48]). It should be noted, however, that the sampling period of each individual in this study is relatively short, and seasonal or annual variations may affect the role of mallards in these phenomena.

### Effects of weather conditions on movement patterns

Wind and precipitation appeared to have no effect on local daily movements of mallards, unlike a study in Switzerland reporting a negative correlation between rain and wind speed with movement activity [27]. Only for the daily number of flights, wind and precipitation were in a candidate model within ΔAICc = 2 from the best model, but additive terms in models within this AICc range should not be interpreted as having any ecological significance [49]. In contrast, temperature contributed to the best fitting models explaining all movement parameters except the number of core areas visited. This is probably related to an increase of foraging activity with rising energetic requirements as temperatures drop, as shown in earlier studies in Europa and North America [25, 26]. Others show high individual variation in the response of mallard movements to variation in temperature [29] and the negative relation between temperature and movement distances may reverse when temperatures drop below 0 °C, as foraging habitat becomes less suitable and birds need to save energy [32, 45].

### Effects of landscape configuration on movement patterns

To explore the mechanisms underlying the large variation in spatial scale of mallard movements between localities, we quantified the configuration of the landscapes with respect to the availability and distribution of surface water. Our data show that regardless of this landscape configuration, mallards depend strongly on the availability of open water. In our study, 95% of the GPS positions were within 38 m from the nearest open water, which is in close agreement with studies in Switzerland (93% within 20 m [29]) and the USA (at least 94% of the time on water [25]). This affinity for water during both day and night, combined with the generally strict separation of roost and foraging sites, results in a strong effect of landscape configuration on the spatial scale of mallard movements. Despite the large variation between individuals, as reported also in other studies [29, 30], mallards clearly had larger home ranges and covered longer distances during commuting flights in drier landscapes in the Netherlands. In very wet landscapes (e.g., Oud Alblas and Terra Nova), commuting movements between roost and foraging sites, defined in the analysis as flights (displacements of >100 m), could even include swimming or walking.

At first glance, the generally lower mobility of mallards at Oud Alblas compared to Terra Nova, which has by far the largest area of surface water, seems counterintuitive. However, mallards tend to avoid large open water bodies, and instead roost on or along shores and forage in shallow water along shorelines (E.K. pers. obs.). The landscape at Oud Alblas is characterized by a dense network of parallel ditches with only 30 to 40 m of wet pastureland in between, offering a wealth of foraging habitat around any roost site. Indeed, across all landscapes (including two other landscapes in the Netherlands, and landscapes in France and Switzerland), flight distances and home range sizes showed a stronger correlation with total length of shoreline than with the total surface area of water. Unfortunately, we had to limit this analysis to home range size and daily maximum flight distance due to differences in tracking methods between studies, but the similarity in responses of all scale-related movement parameters in the analyses of the study sites in the Netherlands suggests that this spatial pattern applies to other movement parameters as well.

The exponential increase of movement distances and home range sizes with a reduction in the total length of shoreline and the number of water bodies in the landscape surrounding the roost implies that mallards will strongly respond to loss of freshwater habitat, even though they are generally considered a highly flexible and opportunistic species. The increased spatial scale of mallard movements in landscapes with more scattered freshwater habitat will help maintain biotic connectivity between isolated wetlands patches, at least up to the degree of fragmentation in the driest landscape used in our study. This may seem good news for the management of wetlands, but we observed an increased tendency to forage on land in drier landscapes and in even drier or more fragmented landscapes mallards might switch to foraging primarily on land, especially in areas where mallards can forage in shallow puddles and on crop waste on agricultural fields [32, 50], thereby reducing their role in connecting wetland biota. The degree of habitat fragmentation at which mallards will completely avoid the landscape is yet to be determined, as well as the relation between habitat fragmentation and the number of mallards and other waterfowl using the landscape. In extremely dry conditions, the relation between waterfowl movements and habitat availability may reverse. In arid Australia, Pacific black ducks (*Anas superciliosa*), a species closely related to the mallard, restrict their movements to the sparsely available freshwater habitat until rainfall-induced floods allow longer exploratory flights [51].

Finally, our finding that water surface area had only a small additive effect on flight distances and did not by itself significantly explain variation in mallard movements, establishes that not the amount of water in a landscape per se is the main factor determining the spatial scale of mallard movements, but rather the availability of foraging habitat (i.e. shallow water along banks and shorelines) in combination with a suitable roost. This relation can be used to predict the spatial scale of mallard movements in landscapes for which no tracking data are available, provided that landscape metrics are calculated at a similarly high spatial resolution as in the present study.

## Conclusions and further implications

The spatial scale of mallard movement behaviour is highly variable between localities differing in their landscape configuration. In particular, the total length of shorelines in the landscape strongly and predictably affects the flight distances and home range size, with weather modulating these movements to a smaller degree. Temporal aspects of movement behaviour were also highly predictable and varied with day length, independent of landscape configuration. Such high predictability of a bird’s spatiotemporal movement pattern suggests that other organisms such as prey, predator and dispersed species, may adapt to optimize their interactions with – and hence, dependency on – these animals. Indeed, mallards may be more of a wetland keystone species than previously thought. The strong, exponential increase of the scale of mallard movement behaviour in landscapes with lower availabilities of freshwater habitat suggests that wetland fragmentation over time will induce a similarly strong response in mallards, leading to longer flight distances. The same could be hypothesized for other waterbird species with strong flight ability [44]. In the case of mallards, their response to environmental change can help maintain the biotic connectivity between isolated habitat patches, at least up to some degree of fragmentation, although our study also shows that mallards are more sensitive to wetland loss and fragmentation than previously considered. The mallard system exemplifies that the effects of habitat loss on the movement ecology of keystone species will have cascading effects on many other organisms living in the same habitat.

## Additional file

Additional file 1:
**Table S1.** Movement parameters per day for mallards in landscapes Oud Alblas (OA), Terra Nova (TN), Juliusput (JP) and Enterveen (EV). Mean values are presented with standard deviation between brackets. **Table S2.** Complete overview of the results of the linear mixed-effects models explaining variation in mallard movement parameters based on Akaike Information Criterion values corrected for small sample size (AICc), difference in AICc from best fitting model (ΔAICc) and Akaike weights (ωi). Model set 1 concerns the analysis of individual flights, model set 2 concerns the analysis of movement metrics per duck day in the Netherlands, and model set 3 concerns the analysis across Western Europe. TOD = time of day (sunrise, day, sunset, night), PC1 (first principal component of PCA on weather parameters) correlates with temperature, PC2 (second principal component) correlates with wind speed and precipitation. Random factors used in the respective models: (1) study site + mallard ID, (2) mallard ID + date, (3) study site + country. **Figure S1.** Patterns of the number of flights (upper panels) and the log-transformed mean flight distance (lower panels) per mallard duck day for the four study areas in the Netherlands (OA = Oud Alblas, TN = Terra Nova, JP = Juliusput, EV = Enterveen) for three scenarios of flight identification, i.e. using threshold values of 100 m, 250 m and 450 m. Displacement >100m was considered a flight in the present study, while 250 m was suggested as a threshold value by Beatty et al. (2014), and 450 m was the maximum distance mallards could cover walking of swimming in a straight line in 15 minutes according to Prange & Schmidt-Nielsen (1970). **Figure S2.** Proportion of flights per half hour relative to the time of sunrise and sunset (left panel), and flight distances per period of the day (right panel), where sunrise and sunset are defined as 1.5 hours before and after sunrise and sunset, respectively, day is the period between sunrise and sunset, and night is the period between the sunrise and sunset periods. Note the log-scale of the right panel y-axis. **Figure S3.** Spatial scale of mallard movements per duck day, represented as the distribution of: (a) flight distances, (b) maximum flight distances, (c) home range sizes (100% MCP), (d) core area sizes (50%KUD), (e) the number of core areas visited, and (f) daily flight frequencies. Note the log-scale of the y-axis of panels c and d. **Figure S4.** Correlations between the landscape parameters water surface area (ha), number of water bodies and total shore length (km) in the study areas in the Netherlands, France and Switzerland. Spearman correlation values and significance levels are indicated (*** indicates p< 0.001). (DOCX 2147 kb)

## References

[1] Bauer S, Hoye BJ (2014). Migratory animals couple biodiversity and ecosystem functioning worldwide. Science.

[2] Nathan R, Getz WM, Revilla E, Holyoak M, Kadmon R, Saltz D, Smouse PE (2008). A movement ecology paradigm for unifying organismal movement research. Proc Natl Acad Sci U S A.

[3] Haig SM, Mehlman DW, Oring LW (1998). Avian movement and wetland connectivity in landscape conservation. Conserv Biol.

[4] Green AJ, Elmberg J (2014). Ecosystem services provided by waterbirds. Biol Rev.

[5] Brochet AL, Guillemain M, Herve F, Gauthier-Clerc M, Green AJ (2009). The role of migratory ducks in the long-distance dispersal of native plants and the spread of exotic plants in Europe. Ecography.

[6] Coughlan NE, Kelly TC, Jansen MAK (2015). Mallard duck (*Anas platyrhynchos*)-mediated dispersal of Lemnaceae: a contributing factor in the spread of invasive *Lemna minuta*?. Plant Biol.

[7] Webster RG, Bean WJ, Gorman OT, Chambers TM, Kawaoka Y (1992). Evolution and ecology of influenza-A viruses. Microbiol Rev.

[8] Olsen B, Munster VJ, Wallensten A, Waldenström J, Osterhaus ADMA, Fouchier RAM (2006). Global patterns of influenza A virus in wild birds. Science.

[9] Darwin C (1859). On the origin of species by means of natural selection, or the preservation of favoured races in the struggle for life.

[10] Figuerola J, Green AJ (2002). Dispersal of aquatic organisms by waterbirds: a review of past research and priorities for future studies. Freshw Biol.

[11] del Hoyo J, Elliot A, Carbot J (1992). Handbook of the birds of the world. Vol 1: Ostrich to Ducks.

[12] Wetlands International (2015). Waterbird population estimates.

[13] Cramp S, Simmons KEL (1977). Handbook of the birds of Europe, the Middle East and North Africa.

[14] Frisch D, Green AJ, Figuerola J (2007). High dispersal capacity of a broad spectrum of aquatic invertebrates via waterbirds. Aquat Sci.

[15] van Leeuwen CHA, van der Velde G, van Lith B, Klaassen M (2012). Experimental quantification of long distance dispersal potential of aquatic smails in the gut of migratory birds. PLoS One.

[16] van Leeuwen CHA, van der Velde G, van Groenendael JM, Klaassen M (2012). Gut travellers: internal dispersal of aquatic organisms by waterfowl. J Biogeogr.

[17] Soons MB, Brochet AL, Kleyheeg E, Green AJ (2016). Seed dispersal by dabbling ducks: an overlooked dispersal pathway for a broad spectrum of plant species. J Ecol.

[18] Kleyheeg E, van Leeuwen CHA, Morison MA, Nolet BA, Soons MB (2015). Bird‐mediated seed dispersal: reduced digestive efficiency in active birds modulates the dispersal capacity of plant seeds. Oikos.

[19] Kleyheeg E, Klaassen M, Soons MB (2016). Seed dispersal potential by wild mallard duck as estimated from digestive tract analysis. Freshw Biol.

[20] van Dijk JGB, Kleyheeg E, Soons MB, Nolet BA, Fouchier RAM, Klaassen M (2015). Weak negative associations between avian influenza virus infection and movement behaviour in a key host species, the mallard *Anas platyrhynchos*. Oikos.

[21] Yamaguchi N, Hiraoka E, Fujita M, Hijikata N, Ueta M, Takagi K (2008). Spring migration routes of mallards (*Anas platyrhynchos*) that winter in Japan, determined from satellite telemetry. Zool Sci.

[22] Bridge ES, Kelly JF, Xiao XM, Takekawa JY, Hill NJ, Yamage M (2014). Bird migration and avian influenza: a comparison of hydrogen stable isotopes and satellite tracking methods. Ecol Indic.

[23] Viana DS, Santamaría L, Michot TC, Figuerola J (2013). Migratory strategies of waterbirds shape the continental-scale dispersal of aquatic organisms. Ecography.

[24] Viana DS, Santamaría L, Michot TC, Figuerola J (2013). Allometric scaling of long-distance dispersal by migratory birds. Am Nat.

[25] Jorde DG, Krapu GL, Crawford RD, Hay MA (1984). Effects of weather on habitat selection and behavior of mallards wintering in Nebraska. Condor.

[26] Legagneux P, Blaize C, Latraube F, Gautier J, Bretagnolle V (2009). Variation in home-range size and movements of wintering dabbling ducks. J Ornithol.

[27] Davis BE, Afton AD (2010). Movement distances and habitat switching by female mallards wintering in the lower Mississippi alluvial valley. Waterbirds.

[28] Link PT, Afton AD, Cox RR, Davis BE (2011). Daily movements of female mallards wintering in southwestern Louisiana. Waterbirds.

[29] Sauter A, Korner P, Fiedler W, Jenni L (2012). Individual behavioural variability of an ecological generalist: activity patterns and local movements of Mallards (*Anas platyrhynchos*) in winter. J Ornithol.

[30] Bengtsson D, Avril A, Gunnarsson G, Elmberg J, Soderquist P, Norevik G (2014). Movements, home-range size and habitat selection of mallards during autumn migration. PLoS One.

[31] Beatty WS, Webb EB, Kesler DC, Raedeke AH, Naylor LW, Humburg DD (2014). Landscape effects on mallard habitat selection at multiple spatial scales during the non-breeding period. Landsc Ecol.

[32] Jorde DG, Krapu GL, Crawford RD (1983). Feeding ecology of mallards wintering in Nebraska. J Wildl Manag.

[33] Webb EB, Smith LM, Vrtiska MP, Lagrange TG (2010). Effects of local and landscape variables on wetland bird habitat use during migration through the rainwater basin. J Wildl Manag.

[34] Pearse AT, Kaminski RM, Reinecke KJ, Dinsmore SJ (2012). Local and landscape associations between wintering dabbling ducks and wetland complexes in Mississippi. Wetlands.

[35] Karelse D, Mandigers F. Blauwgoed, helen en halven: 100 jaar ringwerk in eendenkooien. IJsselstein: Werkgroep Ringwerk Eendenkooien Nederland (WREN); 2013.

[36] Boyd H, Harrison J, Allison A (1975). Mallard *Anas platyrhynchos platyrhynchos*. Duck wings – a study of duck production.

[37] Roshier DA, Asmus MW (2008). Use of satellite telemetry on small-bodied waterfowl in Australia. Mar Freshw Res.

[38] Prange HD, Schmidt-Nielsen K (1970). The metabolic cost of swimming in ducks. J Exp Biol.

[39] Calenge C (2006). The package adehabitat for the R software: a tool for the analysis of space and habitat use by animals. Ecol Model.

[40] Burnham KP, Anderson DR (2002). Model selection and multimodel inference.

[41] Bates D, Maechler M, Bolker B, Walker S (2013). lme4: Linear mixed-effects models using Eigen and S4 R package version 10–4.

[42] Hothorn T, Bretz F, Westfall P (2008). Simultaneous inference in general parametric models. Biom J.

[43] R Core Team (2014). R: a language and environment for statistical computing.

[44] Tamisier A (1979). The functional units of wintering ducks: a spatial integration of their comfort and feeding requirements. Verh Ornithol Ges Bayern.

[45] Paulus SL. Time-activity budgets of nonbreeding Anatidae: a review. In: Waterfowl in winter. Minneapolis: University of Minnesota Press; 1988. p. 135–52.

[46] Cox RR, Afton AD (1996). Evening flights of female northern pintails from a major roost site. Condor.

[47] Kleyheeg E (2015). Seed dispersal by a generalist duck: ingestion, digestion and transportation by mallards (*Anas platyrhynchos*).

[48] van Dijk JGB, Hoye BJ, Verhagen JH, Nolet BA, Fouchier RAM, Klaassen M (2014). Juveniles and migrants as drivers for seasonal epizootics of avian influenza virus. J Anim Ecol.

[49] Arnold TW (2010). Uninformative parameters and model selection using Akaike’s Information Criterion. J Wildl Manag.

[50] Krapu GL, Brandt DA, Cox RR (2004). Less waste corn, more land in soybeans, and the switch to genetically modified crops: trends with important implications for wildlife management. Wildl Soc B.

[51] McEvoy JF, Roshier DA, Ribot RFH, Bennett ATD (2015). Proximate cues to phases of movement in a highly dispersive waterfowl, *Anas superciliosa*. Mov Ecol.

[52] Nakagawa S, Schielzeth H (2013). A general and simple method for obtaining *R*^*2*^ from general linear mixed-effects models. Methods Ecol Evol.

