# Supplementary material for: Movement patterns of a keystone waterbird species are highly predictable from landscape configuration
Source: Mov Ecol. 2017 Feb 1;5:2. doi: 10.1186/s40462-016-0092-7 (PMC5289051; doi:10.1186/s40462-016-0092-7)
Supplement: Additional file 1: — Table S1. Movement parameters per day for mallards in landscapes Oud Alblas (OA), Terra Nova (TN), Juliusput (JP) and Enterveen (EV). Mean values are presented with standard deviation between brackets. Table S2. Complete overview of the results of the linear mixed-effects models explaining variation in mallard movement parameters based on Akaike Information Criterion values corrected for small sample size (AICc), difference in AICc from best fitting model (ΔAICc) and Akaike weights (ωi). Model set 1 concerns the analysis of individual flights, model set 2 concerns the analysis of movement metrics per duck day in the Netherlands, and model set 3 concerns the analysis across Western Europe. TOD = time of day (sunrise, day, sunset, night), PC1 (first principal component of PCA on weather parameters) correlates with temperature, PC2 (second principal component) correlates with wind speed and precipitation. Random factors used in the respective models: (1) study site + mallard ID, (2) mallard ID + date, (3) study site + country. Figure S1. Patterns of the number of flights (upper panels) and the log-transformed mean flight distance (lower panels) per mallard duck day for the four study areas in the Netherlands (OA = Oud Alblas, TN = Terra Nova, JP = Juliusput, EV = Enterveen) for three scenarios of flight identification, i.e. using threshold values of 100 m, 250 m and 450 m. Displacement >100m was considered a flight in the present study, while 250 m was suggested as a threshold value by Beatty et al. (2014), and 450 m was the maximum distance mallards could cover walking of swimming in a straight line in 15 minutes according to Prange & Schmidt-Nielsen (1970). Figure S2. Proportion of flights per half hour relative to the time of sunrise and sunset (left panel), and flight distances per period of the day (right panel), where sunrise and sunset are defined as 1.5 hours before and after sunrise and sunset, respectively, day is the period between sunrise and sunset, and ni [file 40462_2016_92_MOESM1_ESM.docx]

**Supporting Information for manuscript “Movement patterns of a keystone waterbird species are highly predictable from landscape configuration” by Kleyheeg et al.**

Content page

Table S1. 1

Table S2. 2

Figure S1. 4

Figure S2. 5

Figure S3. 6

Figure S4. 7

**Table S1.** Movement parameters per day for mallards in landscapes Oud Alblas (OA), Terra Nova (TN), Juliusput (JP) and Enterveen (EV). Mean values are presented with standard deviation between brackets.

|  |  | **OA** | | **TN** | | **JP** | | **EV** | |
| --- | --- | --- | --- | --- | --- | --- | --- | --- | --- |
| Home range size (ha) | mean | 14.0 | (28.7) | 56.7 | (99.9) | 59.2 | (102.5) | 91.3 | (184.2) |
|  | range | 0.3 - 706.6 | | 0.7 - 611.6 | | 0.5 - 929.0 | | 0.7 - 1416.9 | |
| Core area size (ha) | mean | 0.4 | (0.7) | 1.3 | (2.7) | 1.6 | (2.6) | 2.7 | (5.0) |
|  | range | 0.03 - 16.7 | | 0.03 - 20.6 | | 0.05 - 19.5 | | 0.02 - 40.9 | |
| Max. flight dist. (km) | mean | 0.6 | (0.5) | 1.2 | (1.1) | 1.5 | (1.6) | 2.1 | (2.0) |
|  | max | 0.1 - 5.0 | | 0.1 - 7.5 | | 0.1 - 9.1 | | 0.1 - 14.0 | |
| Mean flight dist. (km) | mean | 0.4 | (0.3) | 0.5 | (0.4) | 0.7 | (0.7) | 0.8 | (0.7) |
|  | max | 0.1 - 1.8 | | 0.1 - 3.3 | | 0.1 - 4.1 | | 0.1 - 4.0 | |
| Number of flights | mean | 3.9 | (1.9) | 6.9 | (3.3) | 6.1 | (3.0) | 6.6 | (3.0) |
|  | range | 0 - 14 | | 0 - 18 | | 0 - 15 | | 2 - 14 | |
| Number of core areas | mean | 1.7 | (0.7) | 2.6 | (1.2) | 2.4 | (1.3) | 2.2 | (1.1) |
|  | range | 1 - 5 | | 1 - 7 | | 1 - 6 | | 1 - 5 | |

**Table S2** Complete overview of the results of the linear mixed-effects models explaining variation in mallard movement parameters based on Akaike Information Criterion values corrected for small sample size (AICc), difference in AICc from best fitting model (ΔAICc) and Akaike weights (ω_i_). Model set 1 concerns the analysis of individual flights, model set 2 concerns the analysis of movement metrics per duck day in the Netherlands, and model set 3 concerns the analysis across Western Europe. TOD = time of day (sunrise, day, sunset, night), PC1 (first principal component of PCA on weather parameters) correlates with temperature, PC2 (second principal component) correlates with wind speed and precipitation. Random factors used in the respective models: (1) study site + mallard ID, (2) mallard ID + date, (3) study site + country.

| Model set | Movement parameters | Independent variables | AICc | ΔAICc | ω_i_ |
| --- | --- | --- | --- | --- | --- |
| (1) | Flight distance | ~ TOD + PC1 | 16394 | 0.00 | 0.65 |
|  |  | ~ TOD | 16396 | 1.49 | 0.31 |
|  |  | ~ TOD + PC1 + PC2 | 16401 | 6.77 | 0.02 |
|  |  | ~ TOD + PC2 | 16402 | 7.71 | 0.01 |
|  |  | NULL | 17705 | 1310.67 | 0.00 |
|  |  | ~ PC2 | 17711 | 1316.57 | 0.00 |
|  |  | ~ PC1 | 17716 | 1321.47 | 0.00 |
|  |  | ~ PC1 + PC2 | 17722 | 1327.52 | 0.00 |
|  |  |  |  |  |  |
| (2) | Number of flights | ~ study site + PC1 | 6790 | 0.00 | 0.73 |
|  |  | ~ study site + PC1 + PC2 | 6792 | 1.99 | 0.27 |
|  |  | ~ study site | 6804 | 13.90 | 0.00 |
|  |  | ~ study site + PC2 | 6804 | 14.82 | 0.00 |
|  |  | ~ PC2 | 6873 | 83.34 | 0.00 |
|  |  | ~ PC1 + PC2 | 6875 | 84.92 | 0.00 |
|  |  | NULL | 6875 | 85.44 | 0.00 |
|  |  | ~ PC1 | 6876 | 86.11 | 0.00 |
|  |  |  |  |  |  |
|  | Mean flight distance | ~ study site + PC1 | 4520 | 0.00 | 1.00 |
|  |  | ~ study site + PC1 + PC2 | 4531 | 11.32 | 0.00 |
|  |  | ~ PC1 | 4542 | 22.55 | 0.00 |
|  |  | ~ study site | 4548 | 28.20 | 0.00 |
|  |  | NULL | 4552 | 32.43 | 0.00 |
|  |  | ~ PC1 + PC2 | 4556 | 36.43 | 0.00 |
|  |  | ~ study site + PC2 | 4562 | 42.15 | 0.00 |
|  |  | ~ PC2 | 4566 | 46.18 | 0.00 |
|  |  |  |  |  |  |
|  | Maximum flight distance | ~ study site + PC1 | 4845 | 0.00 | 1.00 |
|  |  | ~ study site + PC1 + PC2 | 4856 | 10.94 | 0.00 |
|  |  | ~ study site | 4873 | 27.87 | 0.00 |
|  |  | ~ PC1 | 4880 | 34.69 | 0.00 |
|  |  | ~ study site + PC2 | 4886 | 41.53 | 0.00 |
|  |  | NULL | 4890 | 44.95 | 0.00 |
|  |  | ~ PC1 + PC2 | 4893 | 48.29 | 0.00 |
|  |  | ~ PC2 | 4900 | 55.27 | 0.00 |
|  |  |  |  |  |  |
|  | Home range size | ~ study site + PC1 | 4009 | 0.00 | 1.00 |
|  |  | ~ study site + PC1 + PC2 | 4023 | 13.53 | 0.00 |
|  |  | ~ study site | 4033 | 23.57 | 0.00 |
|  |  | ~ study site + PC2 | 4046 | 37.13 | 0.00 |
|  |  | ~ PC1 | 4054 | 44.91 | 0.00 |
|  |  | NULL | 4060 | 50.47 | 0.00 |
|  |  | ~ PC1 + PC2 | 4068 | 59.15 | 0.00 |
|  |  | ~ PC2 | 4072 | 62.64 | 0.00 |
|  |  |  |  |  |  |
|  | Core area size | ~ study site + PC1 | 1032 | 0.00 | 0.99 |
|  |  | ~ study site | 1041 | 8.71 | 0.01 |
|  |  | ~ study site + PC1 + PC2 | 1047 | 14.83 | 0.00 |
|  |  | ~ study site + PC2 | 1057 | 24.65 | 0.00 |
|  |  | NULL | 1068 | 35.79 | 0.00 |
|  |  | ~ PC1 | 1074 | 42.11 | 0.00 |
|  |  | ~ PC2 | 1083 | 50.88 | 0.00 |
|  |  | ~ PC1 + PC2 | 1090 | 58.15 | 0.00 |
|  |  |  |  |  |  |
|  | Number of core areas | ~ study site + PC1 | 4252 | 0.00 | 0.20 |
|  |  | ~ PC2 | 4252 | 0.02 | 0.20 |
|  |  | NULL | 4253 | 0.43 | 0.16 |
|  |  | ~ PC1 | 4253 | 0.85 | 0.13 |
|  |  | ~ PC1 + PC2 | 4253 | 1.01 | 0.12 |
|  |  | ~ study site + PC1 + PC2 | 4254 | 1.44 | 0.10 |
|  |  | ~ study site | 4255 | 2.77 | 0.05 |
|  |  | ~ study site + PC2 | 4256 | 3.17 | 0.04 |
|  |  |  |  |  |  |
| (3) | Maximum flight distance | ~ surface + shore length | 299 | 0.00 | 0.65 |
|  |  | ~ surface + water bodies + shore length | 301 | 1.83 | 0.26 |
|  |  | ~ shore length | 304 | 5.52 | 0.04 |
|  |  | ~ water bodies + shore length | 305 | 6.32 | 0.03 |
|  |  | ~ surface + water bodies | 306 | 7.71 | 0.01 |
|  |  | ~ water bodies | 313 | 14.42 | 0.00 |
|  |  | NULL | 314 | 15.50 | 0.00 |
|  |  | ~ surface | 315 | 16.73 | 0.00 |
|  |  |  |  |  |  |
|  | Home range size | ~ shore length | 480 | 0.00 | 0.47 |
|  |  | ~ surface + shore length | 481 | 1.48 | 0.23 |
|  |  | ~ water bodies + shore length | 481 | 1.51 | 0.22 |
|  |  | ~ surface + water bodies + shore length | 484 | 3.62 | 0.08 |
|  |  | ~ water bodies | 495 | 14.67 | 0.00 |
|  |  | ~ surface + water bodies | 495 | 15.41 | 0.00 |
|  |  | NULL | 499 | 19.37 | 0.00 |
|  |  | ~ surface | 501 | 20.55 | 0.00 |

**
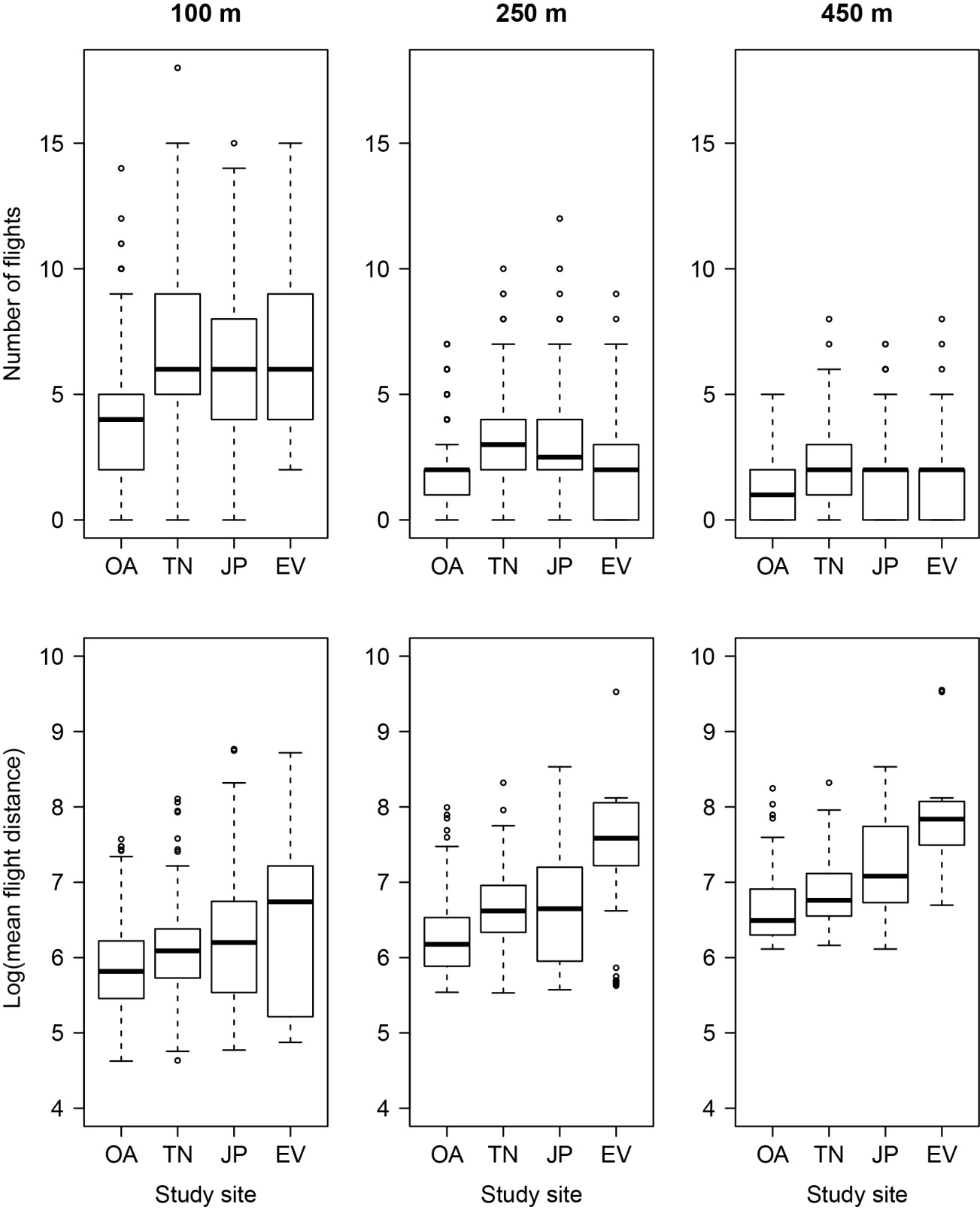
**

**Figure S1. Sensitivity analysis of flight identification threshold**

Patterns of the number of flights (upper panels) and the log-transformed mean flight distance (lower panels) per mallard duck day for the four study areas in the Netherlands (OA = Oud Alblas, TN = Terra Nova, JP = Juliusput, EV = Enterveen) for three scenarios of flight identification, i.e. using threshold values of 100 m, 250 m and 450 m. Displacement >100m was considered a flight in the present study, while 250 m was suggested as a threshold value by Beatty et al. (2014), and 450 m was the maximum distance mallards could cover walking of swimming in a straight line in 15 minutes according to Prange & Schmidt-Nielsen (1970).

**
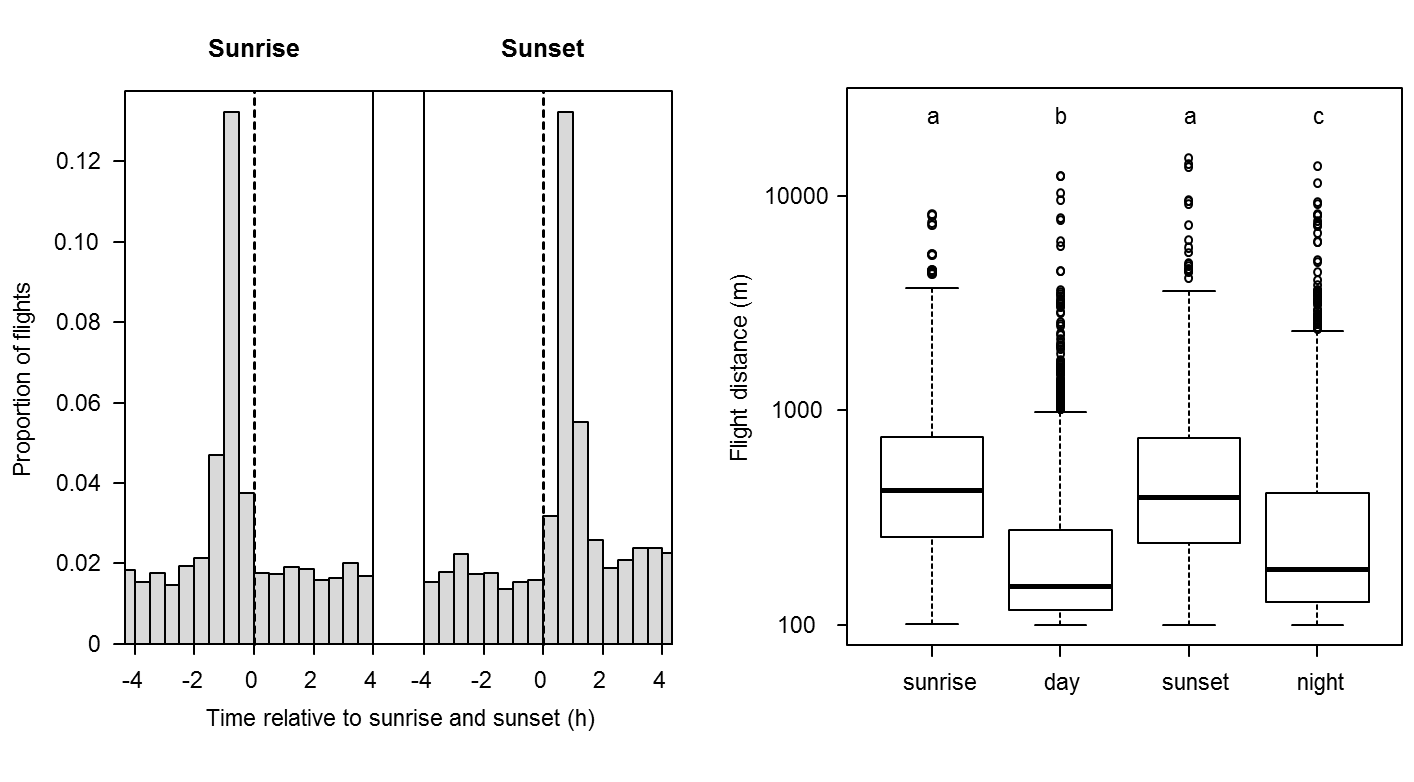
**

**Figure S2. Movement behaviour relative to time of day**

Proportion of flights per half hour relative to the time of sunrise and sunset (left panel), and flight distances per period of the day (right panel), where *sunrise* and *sunset* are defined as 1.5 hours before and after sunrise and sunset, respectively, *day* is the period between sunrise and sunset, and *night* is the period between the *sunrise* and *sunset* periods. Note the log-scale of the right panel y-axis.

**
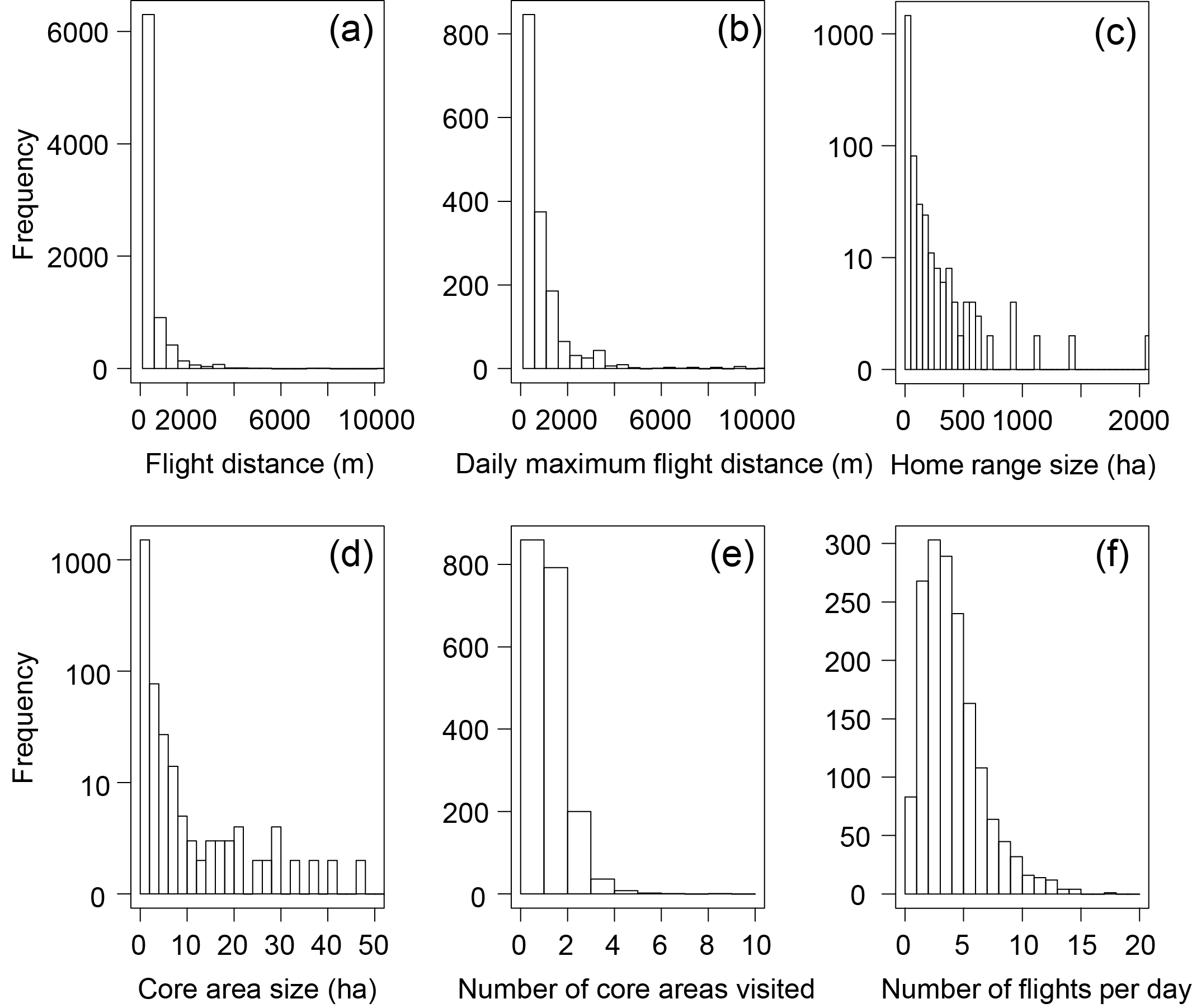
**

**Figure S3. Distribution of general movement parameters**

Spatial scale of mallard movements per duck day, represented as the distribution of: (a) flight distances, (b) maximum flight distances, (c) home range sizes (100% MCP), (d) core area sizes (50%KUD), (e) the number of core areas visited, and (f) daily flight frequencies. Note the log-scale of the y-axis of panels c and d.


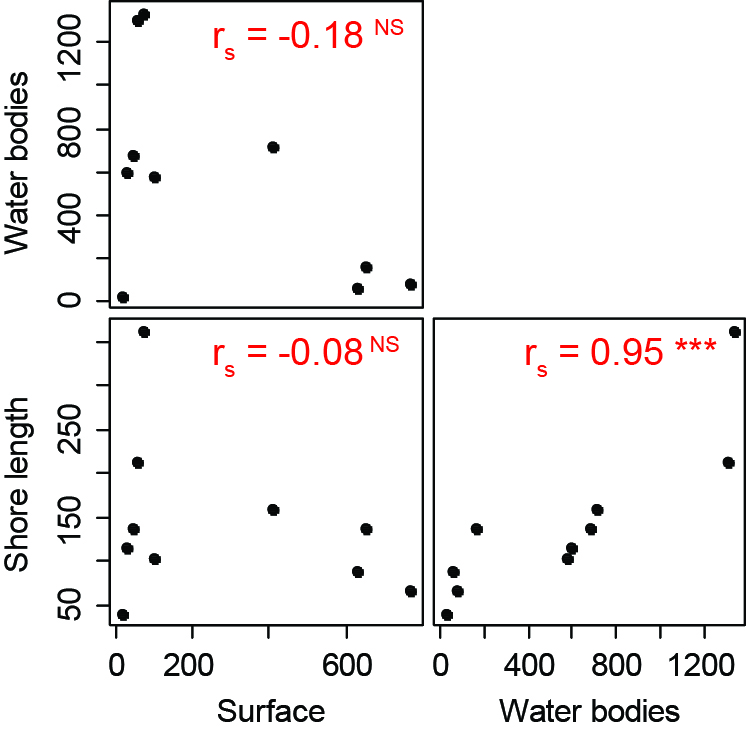


**Figure S4. Correlation between landscape parameters**

Correlations between the landscape parameters water surface area (ha), number of water bodies and total shore length (km) in the study areas in the Netherlands, France and Switzerland. Spearman correlation values and significance levels are indicated (*** indicates *p* < 0.001).
